# In vivo quantification of knee-orthosis valgus-corrective moment during gait: Effects of medial strut length on external knee adduction moment in medial knee osteoarthritis

**DOI:** 10.1371/journal.pone.0337870

**Published:** 2026-08-26

**Authors:** Kosuke Nakano, Yasuhiro Mine, Junji Katsuhira, Kantaro Yamauchi, Masateru Kitashiro, So Nomoto

**Affiliations:** 1 School of Information-Oriented Management, SANNO University, Isehara, Kanagawa, Japan; 2 Faculty of Human Life Design, Toyo University, Kita, Tokyo, Japan; 3 Welfare Engineering Unit, The Polytechnic University of Japan, Kodaira, Tokyo, Japan; 4 Faculty of Design for Welfare Society, Toyo University, Kita, Tokyo, Japan; 5 Keiyu Orthopedic Group, Adachi, Tokyo, Japan; Iran University of Medical Sciences, IRAN, ISLAMIC REPUBLIC OF

## Abstract

Osteoarthritis (OA) is a progressive degenerative disease affecting articular surfaces, and knee orthoses are commonly employed to reduce the external knee adduction moment (EKAM). However, quantitative data on the corrective moments generated by orthoses during gait and their relationship with EKAM remain limited. This study quantified the corrective moments generated by a knee orthosis using a six-axis force sensor and evaluated the impacts of medial lower-leg strut length on these moments and EKAM, aiming to elucidate the biomechanical mechanisms by which orthotic design facilitates unloading in patients with medial knee OA. Sixteen individuals with medial knee OA participated in gait assessments under three conditions: no orthosis and two orthosis configurations differing only in medial lower-leg strut lengths. The same orthosis model was employed for all participants. Only the medial lower-leg strut length was modified between conditions. Kinematic data and ground reaction forces were collected to calculate EKAM, whereas corrective moments were collected concurrently. Because several variables violated normality, nonparametric statistical analyses were employed as appropriate. The 192-mm orthosis generated greater corrective moments than the 152-mm orthosis throughout all the gait phases. During the loading response phase, EKAM was significantly reduced with the 192-mm orthosis compared with both the 152-mm orthosis and no-orthosis cases (p < 0.01). Furthermore, an inverse relationship was observed between the corrective moment and EKAM, indicating that higher orthosis-generated corrective moments were associated with lower EKAM values. Therefore, the medial strut length may be associated with differences in corrective moments and phase-specific reductions in EKAM. These findings indicate that increasing the medial lower-leg strut length may enhance the corrective moment generated by the knee orthoses, leading to phase-specific reductions in EKAM. This suggests that medial strut length is a clinically relevant design parameter for optimizing biomechanical unloading in patients with medial knee OA.

## Introduction

The global acceleration of population aging is contributing to an increasing incidence of bone and joint disorders associated with degenerative changes. In Japan, approximately 36.23 million individuals—representing 29.1% of the total population—are aged 65 years or older, with this proportion expected to increase continuously [1]. Similarly, the World Health Organization projects that the global proportion of individuals aged 60 years or older will increase from 12% in 2015 to 22% by 2050 [2].

As the population ages, the prevalence of knee osteoarthritis (OA) is increasing, affecting 20–38% of individuals aged 65 years or older in developed countries such as Japan and South Korea [3,4]. Knee OA substantially degrades the quality of life by causing chronic pain, restricted mobility, and difficulty performing daily activities, often resulting in loss of independence and increased psychological distress [5,6]. Furthermore, knee OA imposes a substantial economic burden on healthcare systems, as it is a leading cause of disability and frequently necessitates long-term medical management or surgical intervention, including total knee arthroplasty [7,8]. Consequently, noninvasive treatment approaches—such as exercise, weight management, and orthotic intervention—are increasingly recognized as important approaches for managing chronic knee OA [9,10].

Knee OA is a multifactorial disorder influenced by genetic, biological, and mechanical factors, with abnormal mechanical loading recognized as a primary driver of disease onset and progression [11–13]. As the condition advances, patients typically demonstrate increased external knee adduction moment (EKAM) and greater mediolateral joint instability [14,15]. Although conservative treatments, such as exercise and orthotic therapy, are extensively recommended owing to their noninvasive nature [16], exercise alone is insufficient to reduce joint loading, which may contribute to accelerated disease progression [17]. While numerous studies have examined the effects of orthoses on EKAM [18–23], most have focused on changes in EKAM or knee loading under different orthotic conditions, without directly quantifying the valgus corrective moment generated by the device during gait or elucidating its direct relationship with EKAM [18–23].

Recent studies have investigated various orthotic interventions for medial knee OA. For example, Barati et al. [24] conducted a randomized crossover trial comparing a biaxial ankle–foot orthosis (AFO) with a lateral wedge insole. Both devices alleviated pain, enhanced function, and reduced EKAM and its angular impulse; however, the AFO demonstrated superior biomechanical outcomes, albeit with only modest clinical benefits. Similarly, Falahatgar et al. [25] compared a lateral wedge with a subtalar strap (without footwear) to a lateral-wedged insole worn in a sandal. Both devices provided a prompt analgesic effect and yielded comparable EKAM values, with sustained alleviation of pain and improved functional performance after one month of use. Schwarze et al. [26] performed a randomized crossover trial and reported greater load-reducing effects with an AFO than with laterally wedged insoles (approximately 18% versus 6% decrease in the first EKAM peak and 11% versus 5% decrease in the knee adduction angular impulse, KAAI). However, patient-reported outcomes yielded comparable improvements, suggesting no clear clinical superiority. Furthermore, Wang et al. [27] conducted a meta-analysis and observed that foot progression angle modifications influenced EKAM and KAAI. Specifically, toe-in gait reduces the first EKAM peak and KAAI, predominantly in healthy individuals. In contrast, toe-out gait decreases the second EKAM peak and KAAI in patients with medial knee OA. These findings underscore the importance of gait adaptations in modifying medial knee loading. However, both studies lacked precise measurement of the corrective moment generated by knee orthoses during gait, leaving the influence of structural parameters—such as medial strut length—on corrective forces and their relationship with EKAM insufficiently understood.

Therefore, a previous study proposed a method utilizing two force sensors positioned on the medial lower-leg section of the orthosis, targeting the area where load is concentrated under the three-point pressure system, to measure the forces generated by the orthosis [28]. Although this method captured localized forces, it did not elucidate the total corrective moment exerted by the orthosis or its overall effect on EKAM. Subsequent research sought to examine the relationship between the orthotic structure and its corrective forces. For example, one study installed three different orthosis designs on a phantom model and employed strain gauges on structural components to quantify the corrective forces [29]. The results indicated that none of the orthoses generated a sufficient corrective force. However, as the evaluation was conducted on a phantom model, the mechanical characteristics under dynamic conditions during actual gait were not assessed.

Consequently, the magnitude of the valgus corrective moment generated by knee orthoses during ambulation and its influence on EKAM remain unclear. Therefore, experimental studies involving human participants during walking are required. Furthermore, elucidating the relationship between EKAM and the corrective forces exerted by an orthosis necessitates a system capable of quantitatively evaluating the total forces generated by the orthotic structure.

Accordingly, the present study sought to quantify the valgus corrective moment generated by a knee orthosis during gait and to examine its relationship with EKAM. Two primary hypotheses were tested. First, increasing the length of the medial lower-leg strut would enhance the magnitude of the valgus corrective moment generated by the knee orthosis. Second, the relationship between the orthosis-generated valgus corrective moment and EKAM may be influenced by individual gait adaptations and variations in lower-limb alignment. To test these hypotheses, the study measured the actual corrective moment generated by the orthosis, evaluated the impact of medial strut length on the generated moment, and analyzed its association with EKAM during ambulation. The novelty of this study lies in the direct quantification of the valgus corrective moment generated by a knee orthosis during gait using an integrated six-axis force sensor. Furthermore, by altering only the medial lower-leg strut length while maintaining the same orthosis model and size, this study experimentally examined the impact of a specific structural parameter on both the generated corrective moment and EKAM. This approach provides mechanistic insight that extends beyond conventional comparisons of orthosis use versus nonuse.

## Materials and methods

This laboratory-based, repeated-measures experimental study was conducted in the Motion Analysis Laboratory at Toyo University. The study protocol was approved by the Research Ethics Committee of the Graduate School of Life Design at Toyo University (Approval Numbers 2023-A14 and 2024-A6S) and adhered to the principles of the Declaration of Helsinki. Written informed consent was obtained from all participants prior to enrollment. Participant recruitment occurred during two periods: December 6, 2023, to March 31, 2024, and July 22, 2024, to March 31, 2025. Sixteen individuals with left knee OA were enrolled, comprising seven men and nine women. Missing marker trajectory data caused by occlusion were interpolated using the gap-filling function in the Visual3D software package (version 3.6, C-Motion, Inc., Germantown, MD, USA).

Baseline demographics, including age, sex, height, weight, affected side, and disease severity based on the Kellgren–Lawrence (KL) classification, are listed in Table 1. The diagnosis of knee OA was medically confirmed based on the classification criteria of the American College of Rheumatology [30]. Participants were also asked about prior experience with knee braces (yes/no). However, detailed information regarding the duration and frequency of brace use was not collected. Eligibility was restricted to participants with KL grades II and III OA in the left knee, representing mild-to-moderate medial compartment OA for which unloading knee orthoses were clinically indicated. Among the participants, nine had KL Grade III and seven had KL Grade II OA. In all participants, the contralateral right knee was confirmed to be radiographically normal, and participants with bilateral knee OA were not included in the study. To minimize variability owing to side-to-side asymmetry and to ensure standardized testing conditions, only individuals with left-sided knee OA were included in the study. Restricting enrollment to individuals with left-sided knee OA also ensured standardization of the analyzed limb across all participants, facilitating consistent orthosis fitting, marker placement, and interpretation of EKAM and orthosis-generated corrective moments within a unified coordinate and sign convention system. This design method was selected to minimize between-subject variability attributable to side-dependent alignment and gait patterns.

**Table 1:** Characteristics of Patients with Knee Osteoarthritis (OA).

Variable	Patients with Osteoarthritis (n = 16)
Age (years), mean ± SD^a^	70.1 ± 8.44
Sex (n), female/ male	9/ 7
Height (cm), mean ± SD	160.6 ± 8.04
Weight (kg), mean ± SD	70.2 ± 14.6
Affected side (n), left/ right/ bilateral	16/ 0/ 0
Kellgren–Lawrence grade of the observed left knee (n), II/ II	7/ 9

^a^Abbreviation: SD, standard deviation.

Exclusion criteria included the inability to ambulate indoors without assistance, presence of other orthopedic or central nervous system disorders, communication difficulties, epilepsy, fever, alcohol consumption, or significant pain or swelling on the day of measurements.

The sample size for this pilot study was determined using G*Power (version 3.1.9.7, Heinrich Heine University, Düsseldorf, Germany), based on an assumed effect size of 0.3, statistical power of 0.9, an alpha level of 0.05, a correlation among repeated measures of 0.7, and four measurement sessions. These parameters indicated a minimum required sample size of 14 participants [31–33]. For pilot studies lacking robust preliminary evidence, a minimum of 12 participants per group is typically recommended [34]. Accordingly, this study satisfied both the statistical and methodological requirements for a pilot investigation. An anonymized dataset, which does not permit identification of individual participants, is provided as supporting information. Owing to ethical and privacy considerations, access to additional individual-level biomechanical data beyond this dataset may be restricted and will be considered by the corresponding author upon reasonable request.

### Experimental setup and protocol

**Fig 1:**
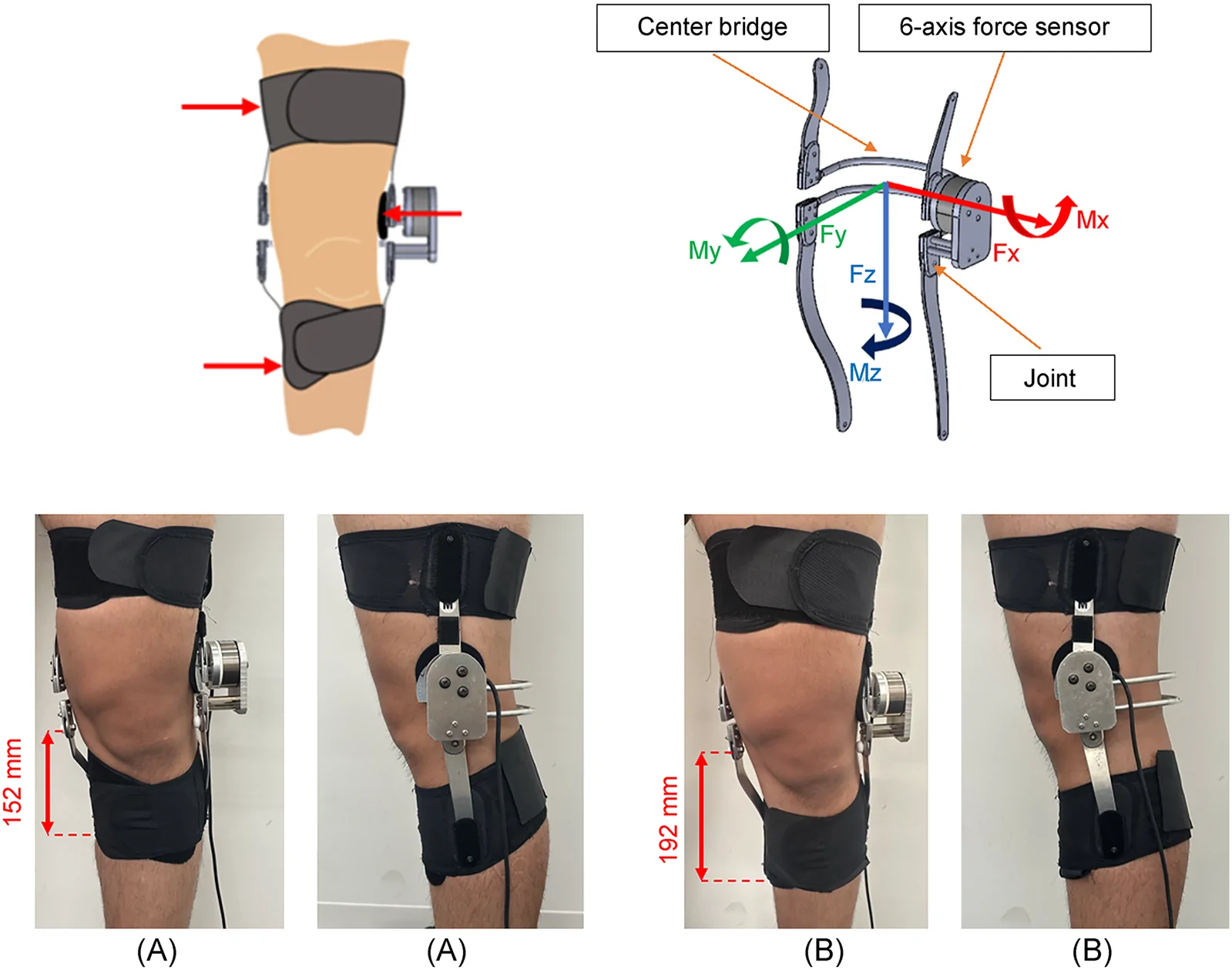
Coordinate framework of the knee orthosis measurement system and orthosis wearing conditions. Knee orthoses with (A) 152 mm and (B) 192 mm medial lower-leg struts.

A measurement system utilizing a center bridge-type knee orthosis (Sakima Prosthetics and Orthotics Co., Ltd., Kunigami District, Okinawa, Japan) evaluated the valgus corrective moment generated by the device. The orthosis delivers valgus correction via a three-point pressure system, providing support at the thigh and lower leg to reduce EKAM (Fig 1).

The center bridge-type knee orthosis was selected for its ability to offer stable three-point support during gait, thereby facilitating accurate measurement of the valgus corrective moment. The orthosis was designed by a certified orthotist. To minimize variability in orthosis configuration during testing, the same principal investigator applied all orthoses and adjusted strap tension. Strap tension was set to achieve firm, yet comfortable fixation, and alignment was standardized based on anatomical landmarks in the frontal plane. Although strap tension, interface compliance, and brace stiffness were not quantitatively measured, the orthosis application procedures were consistently maintained across conditions during evaluations taken by all participants, thereby reducing intra-individual variability.

A six-axis force sensor (Leptrino Co., Ltd., Saku City, Nagano, Japan) was integrated into the lateral rigid joint section of the orthosis to directly quantify the relative forces and varus–valgus moments transmitted between the thigh and shank. In this configuration, load transfer between the thigh and shank occurred exclusively through the sensor mounted on the lateral side, whereas the medial side remained mechanically unconnected to prevent structural interference. This configuration enabled direct measurements of the corrective moment transmitted through the orthosis while preserving the original three-point support mechanism. This sensor simultaneously measures forces and moments along three orthogonal axes, enabling the direct acquisition of relative force and moment data transmitted between the thigh and lower leg.

This configuration facilitates precise mechanical evaluation of the corrective function of the orthosis, a task that is challenging to accomplish using indirect methods, such as conventional gait analysis. The coordinate framework of the six-axis force sensor used a right-handed convention: the x-axis is oriented laterally, the y-axis aligns with the direction of movement, and the z-axis corresponds to the vertical direction, aligned with body weight.

**Fig 2:**
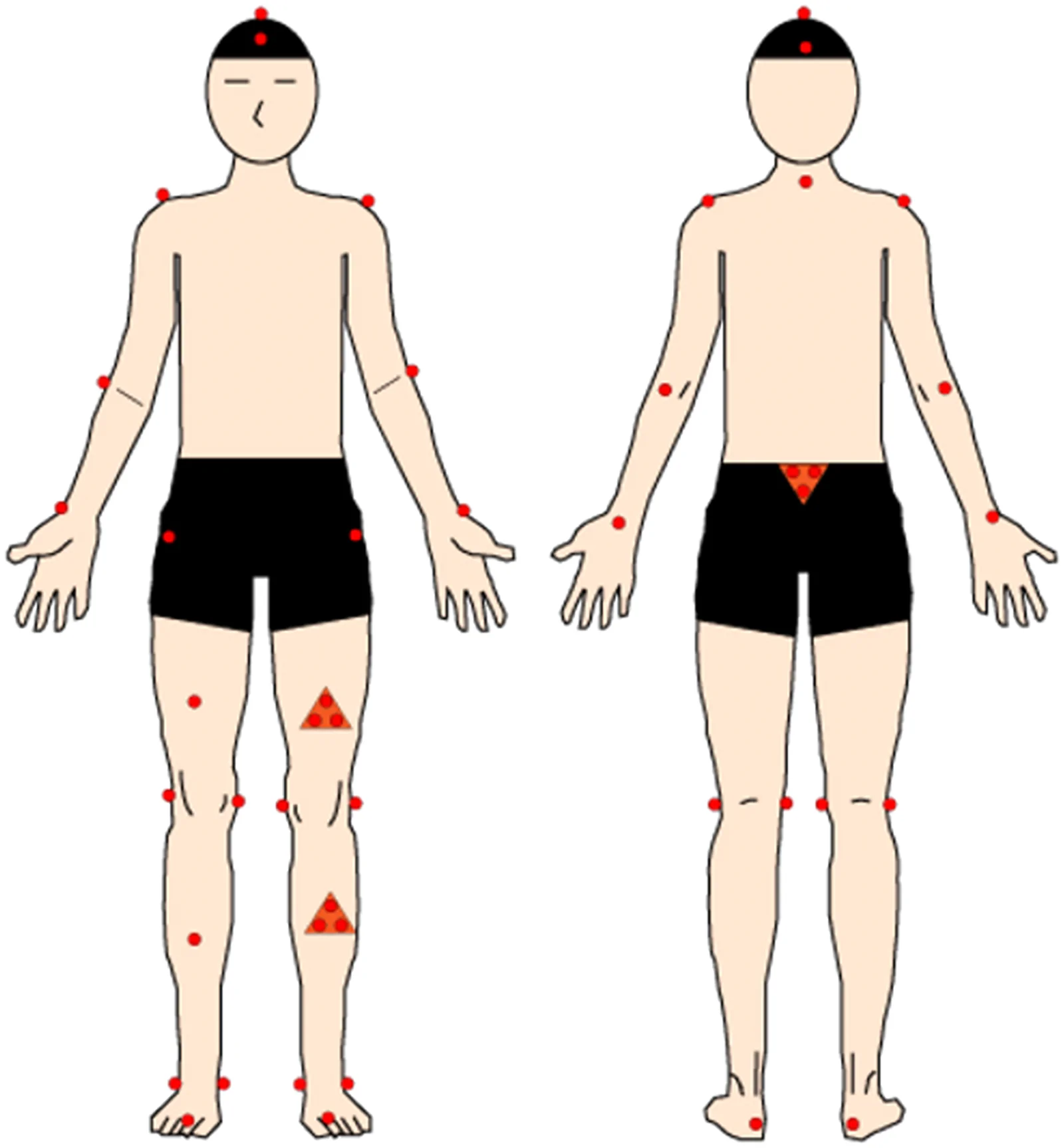
Locations of reflective markers. (A) Anterior and (B) posterior aspects.

The analysis examined the y-axis moment, representing the varus–valgus corrective moment generated by the orthosis. A negative (−) sign indicates that the orthosis applies a valgus (abduction) corrective moment to the lower leg relative to the thigh, whereas a positive (+) sign denotes a varus (adduction) moment. Gait analysis was conducted using seven infrared cameras (Vicon MX T series, Vicon Motion Systems Ltd., Oxford, UK) operated at a sampling frequency of 960 Hz, in conjunction with three force plates (AMTI, Watertown, MA, USA). These systems enabled the simultaneous, high-precision acquisition of three-dimensional body-segment kinematics and ground reaction forces during walking. Reflective markers (14 mm in diameter) were positioned according to the Helen Hayes protocol at key anatomical landmarks: the head (vertex, forehead, and occiput), trunk (seventh cervical vertebra and both acromia), pelvis (anterior and posterior superior iliac spines), upper limbs (elbows and wrists), and lower limbs (thighs, medial and lateral aspects of the knees, shanks, medial and lateral malleoli, and second metatarsals). Furthermore, one auxiliary marker was attached to both the thigh and shank of the left lower limb fitted with the test knee orthosis, resulting in a total of 34 markers for motion capture (Fig 2).

Occasional marker dropouts were observed owing to occlusion by the knee orthosis or interference from surrounding soft tissue. Missing trajectories were interpolated using the spline function in the Vicon Nexus software program (version 2, Vicon Motion Systems Ltd., Oxford, UK), referencing the static standing calibration trial to maintain temporal and spatial consistency within the dataset. Consequently, no gait trials were excluded from the analysis. The participants completed gait testing under three randomized conditions: (1) without an orthosis, (2) with an orthosis featuring a 152 mm medial lower-leg strut, and (3) with an orthosis featuring a 192 mm medial lower-leg strut. The orthosis configurations are shown in Fig 1. For each condition, four gait trials were conducted, yielding a total of 12 trials per participant to ensure sufficient data for reliable analysis. Prior to data collection, participants were allowed to familiarize themselves with walking for 5 min to achieve a natural walking pattern and minimize adaptation effects. Although this familiarization period aimed to reduce short-term adaptation, long-term adaptation differences between habitual and novice brace users were not controlled in this study. Before gait measurements, a 30 s quiet standing posture was recorded in all participants and used as reference data for marker interpolation and for determining the initial alignment of each body segment. Orthosis alignment was visually adjusted by the principal investigator during static standing to approximate the anatomical flexion–extension axis of the knee. However, three-dimensional alignment validation and pressure distribution analysis were not conducted. During gait trials, the reflective markers positioned on the medial, and lateral aspects of the left knee joint were temporarily detached to prevent physical interference with the knee orthosis. Their positions were subsequently reconstructed using the static standing data. This interpolation process, performed in Vicon Nexus, ensured both temporal and spatial consistency across the dataset. All gait measurements were performed on an 8 m flat, straight walkway. Participants were instructed to walk at their preferred, comfortable speed to maintain natural gait characteristics. Throughout each trial, the valgus corrective moment was sampled continuously using an integrated six-axis force sensor. At the initiation of each measurement, the sensor output was calibrated to a zero moment (0 N·m) under the no-orthosis condition, enabling extraction of the corrective moment generated by the orthosis during gait. The six-axis force sensor (SAN050102200A00, Leptrino Co., Ltd., Saku, Nagano, Japan) has a rated moment capacity of ±20 N·m, with nonlinearity and crosstalk outcomes within ±1.0% and ±2.0% of the rated output, respectively. The sensor was factory-calibrated prior to integration into the orthosis, and zero-offset calibration was conducted under unloaded conditions before each measurement session.

### Data analysis

Kinematic and kinetic data were processed using Visual3D (version 3.6, C-Motion, Germantown, MD, USA). The acquired raw marker data were filtered using a low-pass Butterworth filter at a cutoff frequency of 6 Hz. Similarly, raw data acquired from the ground reaction forces and the Leptrino six-axis force sensor, which is integrated into the knee orthosis measurement system, were filtered using a low-pass Butterworth filter with a 15 Hz cutoff frequency. All gait data were time-normalized to represent 100% of the stance phase. Knee joint moments and gait performance parameters, including walking speed and stride length, were subsequently computed. Both the knee joint and valgus corrective moments generated by the knee orthosis, as measured by the six-axis force sensor, were normalized by the participants’ body weights.

The analysis focused on the stance phase of the gait cycle, during which substantial mechanical loading was observed at the knee joint. Based on the vertical component of the ground reaction force, the stance phase was subdivided into three subphases: loading response, mid- and terminal stance, and pre-swing. The loading response phase was defined as the period between the initial contact of the affected (left) limb and toe-off of the unaffected right lower limb. The mid and terminal stance phases encompassed the period from toe-off to the subsequent initial contact of the unaffected right lower limb. The pre-swing phase was defined as the period between the initial contact of the unaffected right lower limb and the toe-off of the affected limb.

Integrated values of the EKAM and valgus corrective moment generated by the orthosis were calculated under three test conditions: no orthosis, orthosis with a 152 mm medial lower-leg strut, and orthosis with a 192 mm strut. These values were determined for all the subphases—loading response, mid- and terminal stance, and pre-swing—as well as for the entire stance phase. Walking speed and stride length were also analyzed under the same three conditions to assess gait characteristics.

### Statistical analyses

Statistical analyses were conducted using the JASP software package (version 0.19.3.0, JASP Team, Amsterdam, The Netherlands). The alpha threshold was set at *p* < 0.05, and all tests were two-tailed. The normality of EKAM, valgus corrective moment generated by the orthosis, walking speed, and stride length were evaluated using the Shapiro–Wilk test (*p* < 0.05). Parametric tests were performed when the assumption of normality was satisfied; otherwise, nonparametric methods were utilized. Walking speed and stride length were compared across three test conditions: (1) no orthosis, (2) orthosis with a 152 mm medial lower-leg strut, and (3) orthosis with a 192 mm strut. As these data failed to satisfy the normality assumption, the Friedman test—a nonparametric repeated-measures method—was applied. When significant differences were identified, pairwise comparisons were performed using Conover’s post-hoc test, with Holm’s correction applied to control the family-wise error. EKAM was analyzed under the same three conditions using the same procedure. However, for the pre-swing phase of EKAM, which satisfied the normality assumption, a parametric repeated-measures analysis of variance (ANOVA) was conducted. Effect sizes were calculated as Kendall’s W for the Friedman tests and as η² for repeated-measures ANOVA. According to Cohen’s guidelines, W values of 0.1, 0.3, and 0.5 were interpreted as small, moderate, and large effects, respectively, whereas η² values of 0.01, 0.06, and 0.14 indicated small, moderate, and large effects, respectively [35].

To directly compare the orthoses with 152 mm and 192 mm medial lower-leg struts, the Wilcoxon signed-rank test—a nonparametric test well-suited for paired data—was employed. Effect sizes for the Wilcoxon tests were reported as r values, calculated by dividing the z statistic by the square root of the sample size (r = z/ √n). According to Cohen’s benchmarks, |r| = 0.1, 0.3, and 0.5 were interpreted as small, moderate, and large effects, respectively [35]. Finally, Spearman’s rank correlation coefficient was used to evaluate the relationship between the orthosis-generated valgus corrective moment and EKAM under both the 152 mm and 192 mm strut conditions. This nonparametric approach was selected owing to its minimal assumptions regarding linearity and its robustness to outliers, making it particularly suitable for small-sample clinical datasets with high variability [36]. The strength of the correlations adhered to Cohen’s criteria [35]: |r| > 0.1 was considered a weak correlation, |r| > 0.3 a moderate correlation, and |r| > 0.5 a strong correlation. To further evaluate the robustness of the correlation estimates, 95% confidence intervals were calculated using a nonparametric bootstrap method with 5000 resamples. Furthermore, a leave-one-out sensitivity analysis was conducted, whereby the Spearman correlation coefficient was recalculated 16 times (by excluding one participant each time). The range of coefficients across all iterations was assessed to evaluate the influences of individual cases.

## Results

### Gait performance

The gait speed and stride length for all orthotic conditions are listed in Table 2. The mean gait speed without an orthosis was 1.09 ± 0.16 m/s. With the 152 mm medial lower-leg strut orthosis, gait speed was 1.10 ± 0.12 m/s, indicating no significant differences compared with either the no-orthosis or the 192 mm orthosis condition (p = n.s.). In contrast, the mean gait speed with the 192-mm orthosis was 1.07 ± 0.13 m/s, which was significantly lower than that observed with the 152-mm orthosis (*p* < 0.05). Stride length remained consistent across all three conditions, with mean values of 1.19 ± 0.08 m (no orthosis), 1.19 ± 0.06 m (152 mm orthosis), and 1.18 ± 0.07 m (192 mm orthosis).

**Table 2:** Comparisons of Gait Speed and Stride Length in Patients with Knee OA Across Three Conditions: No Orthosis, 152-mm Strut Orthosis, and 192-mm Strut Orthosis.

Variable		Patients with OA
**Gait speed (m/s)**	Without orthosis, mean ± SD	1.09 ± 0.16
	152 mm medial lower leg strut orthosis, mean ± SD	1.10 ± 0.12
	192 mm medial lower leg strut orthosis, mean ± SD	1.07 ± 0.13^*^
**Stride length (m)**	Without orthosis, mean ± SD	1.19 ± 0.08
	152 mm medial lower leg strut orthosis, mean ± SD	1.19 ± 0.06
	192 mm medial lower leg strut orthosis, mean ± SD	1.18 ± 0.07

Note: * *p* < 0.05 compared with the 152 mm strut orthotic condition.

### EKAM reduction

**Fig 3:**
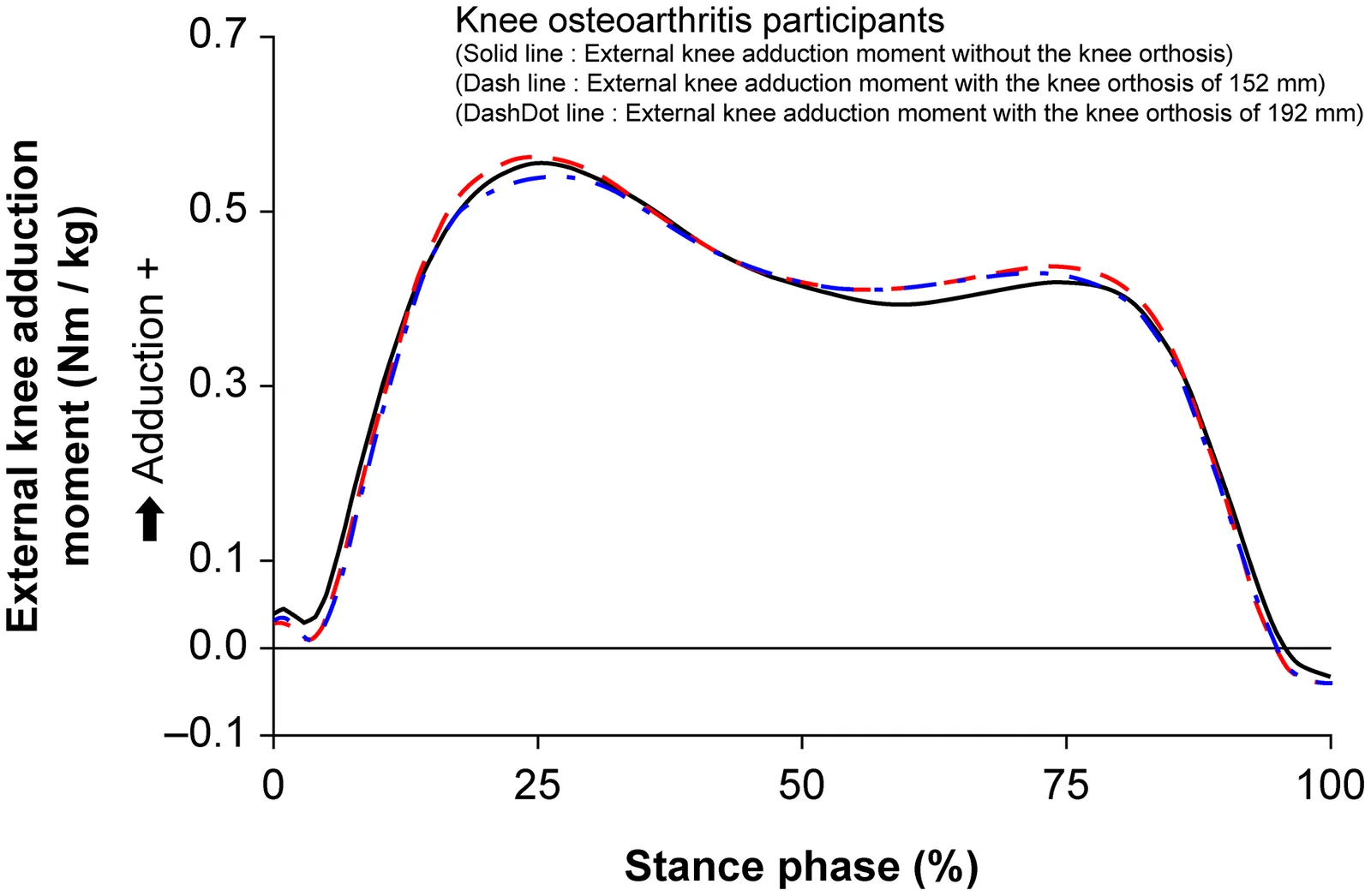
Knee adduction moment in ambulation with and without knee orthoses equipped with 152 mm and 192 mm medial lower-leg struts.

The EKAM in patients with knee OA was evaluated across the three experimental conditions: no orthosis, 152 mm medial lower-leg strut orthosis, and 192 mm strut orthosis. The results are listed in Table 3 and presented in Fig 3.

**Table 3:** Comparison of the External Knee Adduction Moment (EKAM) in Patients with Knee OA Under No-orthotic Conditions and Conditions with Orthoses with Different Medial Lower-leg Strut Lengths.

Variable	Patients (without orthosis) Mean ± SD (Nm·s/ kg)	Patients (with 152 mm medial lower leg strut orthosis) Mean ± SD (Nm·s/ kg)	Patients (with 192 mm medial lower leg strut orthosis) Mean ± SD (Nm·s/ kg)	Test^a^	Effect size^b^	Post-hoc Comparisons (Holm-adjusted)^c^
EKAM during loading response	0.022 ± 0.012	0.019 ± 0.013	0.015 ± 0.011	Friedman	W = 0.27	No vs. 152 mm orthosis **, No vs. 192 mm orthosis **, 152 mm orthosis vs. 192 mm orthosis **
EKAM during mid and terminal stance	0.205 ± 0.064	0.209 ± 0.068	0.213 ± 0.068	Friedman	W = 0.04	n.s.^d^
EKAM during pre-swing	0.019 ± 0.011	0.018 ± 0.010	0.017 ± 0.010	RM-ANOVA^e^	η² = 0.00	n.s.
EKAM during stance phase	0.245 ± 0.081	0.245 ± 0.084	0.244 ± 0.084	Friedman	W = 0.00	n.s.

Note: **p < 0.01.

^a^Omnibus comparisons were performed using Friedman tests or RM- ANOVA as indicated in the “Test” column.

^b^Effect sizes are reported as Kendall’s W values for Friedman tests and η² values for RM-ANOVA.

^b^When significant, pairwise comparisons were conducted with Holm-adjusted post-hoc tests.

^d^Comparisons without asterisks indicate no significant difference (n.s.).

^e^Abbreviation: RM-ANOVA, repeated-measures analysis of variance.

During the loading response phase, EKAM was significantly reduced with orthotic intervention. The time-integrated EKAM without an orthosis was 0.022 ± 0.012 Nm·s/kg, which decreased significantly to 0.019 ± 0.013 Nm·s/kg with the 152 mm strut (*p* < 0.01). Similarly, with the 192 mm strut, EKAM was further reduced to 0.015 ± 0.011 Nm·s/kg, a value significantly lower than both the no-orthosis (p < 0.01) and 152 mm strut (p < 0.01) conditions. However, during the mid- and terminal stance and pre-swing phases, the time-integrated EKAM values were consistent across all conditions, and no significant differences were observed. Furthermore, the time-integrated EKAM values across the full stance phase were 0.245 ± 0.081, 0.245 ± 0.084, and 0.244 ± 0.084 Nm·s/kg for the no orthosis, 152 mm, and 192 mm orthoses, respectively, indicating no significant differences among the three conditions (*p* > 0.05). These findings suggest that the observed time-integrated EKAM reduction was limited to the loading response phase.

Across the stance phase, larger valgus corrective moments were observed in phases with relatively higher EKAM values, indicating a potential phase-dependent association between the two variables.

### Valgus corrective moment

**Fig 4:**
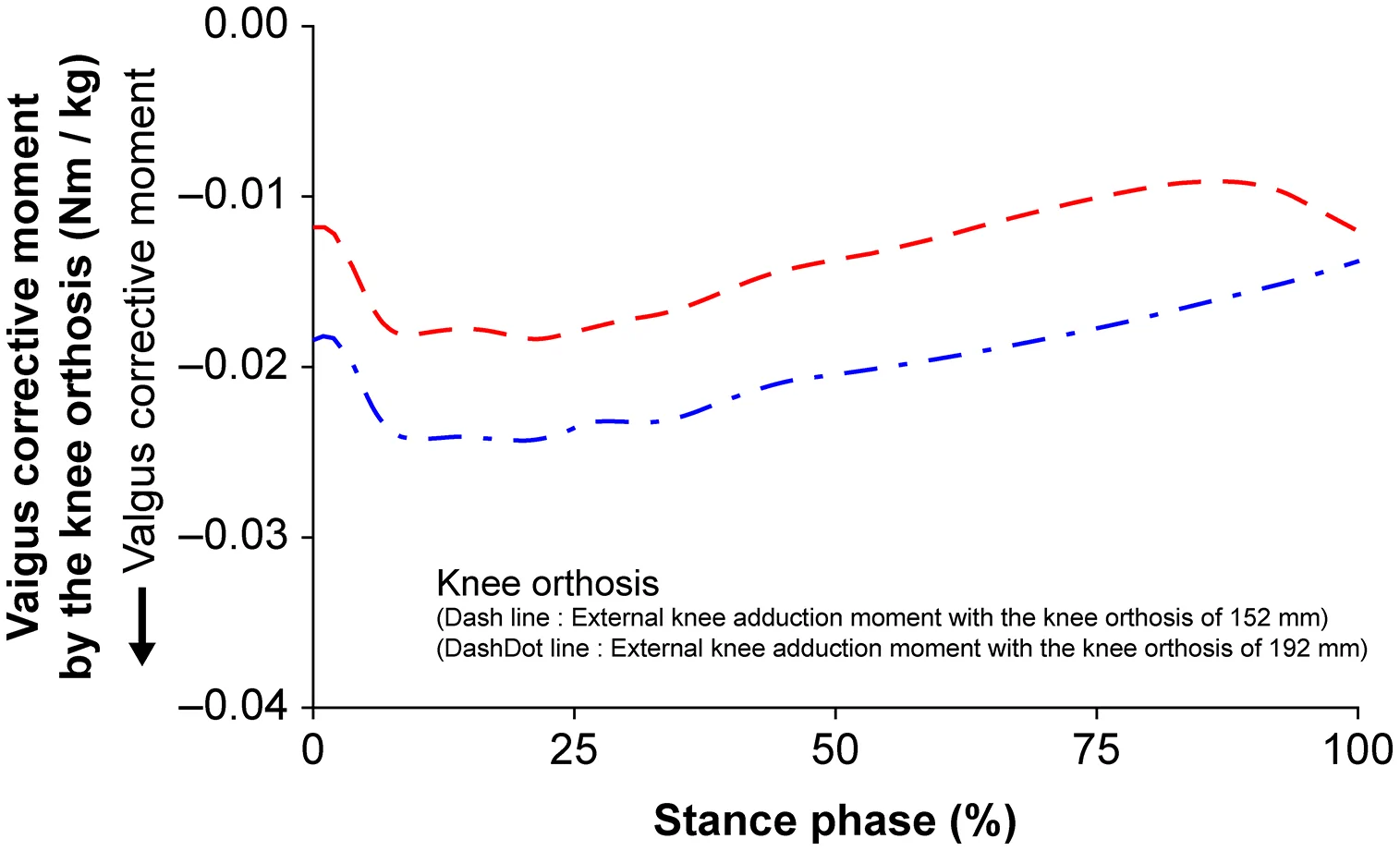
Valgus corrective moment generated by knee orthoses with different medial lower-leg strut lengths.

The valgus corrective moment generated by the knee orthosis was influenced by the length of the medial lower-leg strut (Fig 4). Across the stance phase, the orthosis with the 192 mm strut consistently generated a greater valgus corrective moment than that generated with the 152 mm strut. The corrective moment demonstrated a pronounced initial increase during the loading response phase.

The significant differences in corrective moments between the two orthosis configurations are listed in Table 4. During the loading response phase, the corrective moments were −0.0017 ± 0.00068 and −0.0021 ± 0.00089 Nm·s/kg for the 152 mm and 192 mm struts, respectively; these results are significantly different *(p* < 0.01)*.* A similar pattern was observed during the mid- and terminal stance phases, in which the corrective moments were −0.0063 ± 0.0027 Nm·s/kg and −0.0097 ± 0.0036 Nm·s/kg for the 152 mm and 192 mm struts, respectively (*p* < 0.01).

**Table 4:** Comparison of Valgus Corrective Moments Generated by the 152 mm and 192 mm Medial Lower-leg Strut Orthoses.

Variable	Valgus Corrective Moment Generated by the 152 mm Medial Lower Leg Strut Orthosis Mean ± SD (Nm·s/ kg)	Valgus Corrective Moment Generated by the 192 mm Medial Lower Leg Strut Orthosis Mean ± SD (Nm·s/ kg)	*P*-value	Effect Size (r)
Corrective moment during loading response	−0.0017 ± 0.0007	−0.0021 ± 0.0009	**	0.49
Corrective moment during mid and terminal stance	−0.0063 ± 0.0027	−0.0097 ± 0.0036	**	0.86
Corrective moment during pre-swing	−0.0012 ± 0.0008	−0.0018 ± 0.0009	**	0.73
Corrective moment during stance phase	−0.0092 ± 0.0037	−0.014 ± 0.0049	**	0.83

Note: Statistical significance is indicated as * *p* < 0.05 and ** *p* < 0.01.

During both the pre-swing and entire stance phases, the 192 mm strut orthosis consistently demonstrated a significantly larger valgus corrective moment than that of the 152 mm configuration (*p* < 0.01). During the pre-swing phase, the corrective moment was −0.0012 ± 0.00075 Nm·s/kg for the 152 mm strut and −0.0018 ± 0.00085 Nm·s/kg for the 192 mm strut. Over the entire stance phase, the integrated corrective moments were equal to −0.0092 ± 0.0037 and −0.014 ± 0.0049 Nm·s/kg, respectively. Overall, the 192 mm orthosis generated significantly greater valgus corrective moments across all the subphases of the stance phase (*p* < 0.01).

### Relationship between the valgus corrective moment generated by a knee orthosis and EKAM

**Fig 5:**
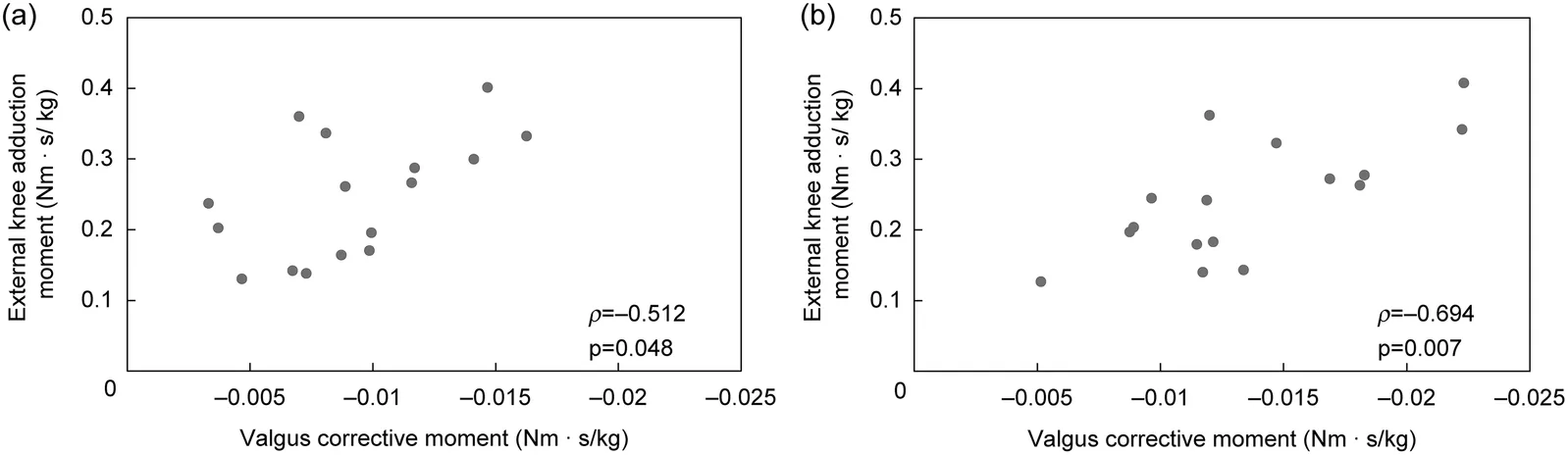
Association between the EKAM and valgus corrective moment generated by the knee orthosis under the following tested conditions: (A) 152 mm and (B) 192 mm medial lower-leg strut orthoses.

The relationship between the valgus corrective moment generated by the knee orthosis and EKAM under the 152 mm medial lower-leg strut condition is shown in Fig 5A. A significant negative correlation was observed between the two variables, as indicated by Spearman’s rank correlation coefficient (ρ = −0.512, *p* = 0.048). According to Cohen’s criteria [35], this correlation represents a moderate association between the corrective moment and EKAM, with the coefficient slightly exceeding 0.50 and approaching the threshold for a strong correlation.

The corresponding relationship under the 192 mm medial lower-leg strut condition demonstrated a stronger negative correlation (ρ = −0.694, *p* = 0.007). Based on Cohen’s criteria, this correlation qualifies as a strong association, indicating that the magnitude of EKAM was more closely associated with variations in the corrective moment. Bootstrap analysis (5000 resamples) yielded 95% confidence intervals of −0.873 to −0.009 for the 152 mm condition and −0.910 to −0.278 for the 192 mm condition. In both cases, the confidence intervals did not include zero, supporting the stability of the observed associations. The leave-one-out sensitivity analysis further demonstrated that the correlation coefficient ranged from −0.418 to −0.639 for the 152 mm condition and from −0.629 to −0.761 for the 192 mm condition. The direction of association remained consistently negative across all iterations, indicating that the observed correlations were not driven by any single influential participant.

These findings indicate that for both orthosis configurations, increases in the valgus corrective moment generated by the orthosis were typically associated with decreases in EKAM. The stronger correlation observed with the 192 mm strut suggests that a longer medial lower-leg strut may be associated with greater reductions in EKAM.

## Discussion

This study addressed two hypotheses. First, extending the medial lower-leg strut of the knee orthosis would increase the valgus corrective moment generated by the orthosis. Second, the relationship between the orthosis-generated corrective moment and EKAM may be influenced by factors such as changes in lower-limb alignment and gait patterns. Unlike previous studies that only compared orthosis use versus nonuse, this study directly quantified the corrective moment using a six-axis force sensor integrated into the orthosis. This approach enabled, for the first time, a direct evaluation of the relationship between the measured corrective moment and EKAM during walking. The findings provide novel biomechanical insights into how structural variations in knee orthoses—specifically differences in medial strut length—affect the generated corrective moment and its impact on EKAM.

The first hypothesis was partially supported by the data. Specifically, the 192 mm strut orthosis consistently generated significantly greater valgus corrective moments throughout the stance phase (*p* < 0.01). This increase can be attributed to the longer moment arm from the strut tip to the knee joint’s center, which theoretically generates a larger moment under an equivalent reaction force. However, the effective in vivo moment arm was not directly measured or geometrically quantified in this study; therefore, this mechanical interpretation remains inferential rather than experimentally validated. In this study, the 192 mm strut orthosis demonstrated higher corrective moments compared with the 152 mm orthosis, particularly during the early loading response phase. These findings highlight the critical role of orthotic structural parameters during the loading response phase, providing valuable insights for the design of moment-generating mechanisms in knee orthoses. Similar trends have been observed in previous studies. For example, Pua et al. [37] demonstrated that even when vertical orthosis dimensions were identical, variations in the relative moment arm—resulting from differences in user height and leg length—influenced orthotic performance. Their findings indicated that shorter participants experienced greater reductions in EKAM [37], suggesting that a longer relative moment arm enhances the generated corrective moment. Collectively, these findings indicate that extending the strut length enhances the production of corrective moments, highlighting the strut length as a critical design parameter in the development of orthoses.

The observed relationship between orthotic structure and EKAM reduction aligns with previous research findings, demonstrating that valgus knee bracing modifies and typically decreases EKAM during gait [18,20]. Structural variations—including differences in orthosis–insole combinations and overall brace configuration—influence both kinematic and kinetic responses [21,22], thereby influencing the biomechanical effectiveness of the orthosis [23]. Building on prior work that compared corrective forces across various orthosis types, this study extends the understanding from observed effects to underlying mechanisms by directly measuring the orthosis valgus-corrective moment [29]. The findings indicate that medial strut length significantly influences the magnitude of the generated corrective moment and its relationship with EKAM. Furthermore, minor gait adaptations, such as slight reductions in walking speed, were observed and are consistent with previous reports that anthropometric and spatiotemporal factors can influence bracing effects and EKAM magnitude [37,38].

These results are consistent with those of Schwarze et al. [26], who reported greater reductions in EKAM with an AFO compared with laterally wedged insoles, despite similar clinical outcomes between devices. Similarly, Barati et al. [24] observed that both AFOs and lateral wedges reduced EKAM, with AFOs providing greater biomechanical improvements but without corresponding differences in clinical outcomes. In contrast, Falahatgar et al. [25] observed no significant variations in EKAM between orthotic designs, despite symptomatic improvements, highlighting the importance of orthotic structures in biomechanical efficacy. Furthermore, the gait adaptations observed in this study are consistent with the findings of Wang et al. [27], whose meta-analysis demonstrated that modifications in the foot progression angle can differentially reduce EKAM peaks, suggesting a potential interaction between orthosis-generated forces and user-specific gait adaptations.

The second objective was to investigate the association between the valgus corrective moment generated by the knee orthosis and EKAM. The findings provided partial support for this hypothesis. During the loading response phase, both the 152 and 192 mm strut orthoses yielded significant reductions in EKAM compared with the no-orthosis condition. Furthermore, the 192 mm strut orthosis achieved a significantly greater reduction in EKAM than the 152 mm orthosis. However, during the mid- and terminal stance phases, no significant difference in EKAM was observed between the orthotic and non-orthotic conditions. These findings suggest that the corrective effect of the orthosis is primarily concentrated in the loading response phase, aligning with previous studies [18,20] that have demonstrated the phase-specific influence of knee orthoses during the gait cycle. Because the loading response phase represents a critical period of medial knee joint loading, the observed reduction in EKAM during this phase may have biomechanical implications for patients with knee OA. In contrast, during the mid and terminal stance phases, EKAM yielded slightly higher mean values under the orthosis conditions (0.205 ± 0.064 Nm·s/kg without orthosis, 0.209 ± 0.068 Nm·s/kg with the 152 mm orthosis, and 0.213 ± 0.068 Nm·s/kg with the 192 mm orthosis), although these differences were not significant. Similarly, during the pre-swing phase, minor differences were observed (0.019 ± 0.011, 0.018 ± 0.010, and 0.017 ± 0.010 Nm·s/kg, respectively); however, none reached statistical significance. Because no distinct decrease in EKAM was observed during the later stance phases, the reduction observed during the loading response phase did not translate into a significant difference in the time-integrated EKAM across the entire stance phase. This suggests that the reduction in EKAM during the loading response phase may have been partially offset by the slight, insignificant increases observed during the later stance phases. The second hypothesis posited that the relationship between the valgus corrective moment generated by the orthosis and EKAM would be weak, owing to potential confounding factors, such as lower-limb alignment and gait pattern alterations. Contrary to this expectation, the results demonstrated a significant negative correlation between the valgus corrective moment generated by the orthosis and EKAM (*p* < 0.05).

Previous studies have primarily evaluated changes in EKAM or knee loading under different orthotic conditions using inverse dynamics [18–23]. Consequently, the relationship between the corrective moment generated by the orthosis and EKAM has not been fully elucidated. To address this gap, the present study directly measured the corrective moment during gait, enabling quantitative analysis of its relationship with EKAM. A significant negative correlation was observed in both cases. For the 152 mm strut, ρ = −0.512 (*p* < 0.05), whereas for the 192 mm strut, ρ = −0.694 (*p* < 0.01), indicating a stronger association with the longer strut. According to Cohen’s criteria, a correlation coefficient of ρ = −0.512 denotes a moderate correlation, whereas ρ = −0.694 reflects a strong correlation. These findings indicate a moderate-to-strong negative association between the orthosis-generated corrective moment and EKAM during the loading response phase. Specifically, individuals who generated larger valgus corrective moments demonstrated lower time-integrated EKAM values under identical experimental conditions. Notably, EKAM is a surrogate biomechanical indicator derived from inverse dynamics and does not directly quantify intra-articular contact forces, pain, or functional performance. Therefore, the observed association should be interpreted strictly as evidence of mechanical load modulation, rather than as confirmation of symptomatic or functional improvements. Furthermore, patient-reported outcomes, perceived comfort, and longer-term adaptation effects were not assessed in this study; therefore, the clinical implications of increased corrective moment magnitude remain speculative. Notably, significant reductions in EKAM were observed only during the loading response phase, with no significant differences detected during the combined mid and terminal stance phase, the pre-swing phase, or across the entire stance phase. This suggests that the unloading effect is temporally limited rather than sustained throughout the stance phase.

Gait speed and stride length were compared across three conditions: no orthosis, 152 mm strut orthosis, and 192 mm strut orthosis. Gait speed was significantly lower with the 192 mm strut condition compared with that under the 152 mm strut condition. This reduction may be attributed to the increased valgus corrective moment associated with the longer strut, which may introduce greater mechanical constraint at the knee joint and prompt subtle adjustments in gait rhythm.

In contrast, stride length did not differ significantly among the conditions, indicating that strut length had minimal impact on spatial gait parameters. However, a trend toward shorter stride lengths was observed with greater orthotic correction conditions, suggesting that the mechanical intervention of the knee orthosis may have induced minor adaptations in the walking pattern. Collectively, these gait characteristics suggest that the observed reduction in gait speed was likely attributable primarily to changes in temporal parameters, such as cadence, rather than spatial parameters, such as stride length.

A similar pattern was reported by Pollo et al. [20], who demonstrated that valgus knee orthoses significantly decreased EKAM and slightly reduced gait speed. Furthermore, Lusardi et al. [39] highlighted that both the physical and psychological demands of orthosis use can influence voluntary walking speed, supporting the present observations. Furthermore, gait speed influences EKAM magnitude; Astephen et al. [38] demonstrated that lower walking speeds are often associated with reduced EKAM values.

Collectively, these findings suggest that the modest reduction in gait speed observed with the 192 mm strut orthosis may have contributed to the corresponding decrease in EKAM, alongside the increased valgus corrective moment generated by the orthosis. However, gait speed was neither controlled for nor included as a covariate in the statistical analyses. Therefore, the extent to which the observed reduction in EKAM is attributable to the increased corrective moment versus speed-related gait adaptations remains unclear. Future research should incorporate gait speed as a covariate or standardize walking speed to elucidate this relationship. Importantly, the degree of speed reduction remained within clinically acceptable limits and would unlikely impair daily activities. From a clinical perspective, optimizing medial strut length may enhance the mechanical corrective force while minimizing gait-related effects. However, whether these biomechanical changes translate into meaningful reductions in pain, improvements in function, or long-term disease progression remains to be established in future clinical studies. Furthermore, this study has both educational and practical significance. To our knowledge, this is the first study that directly quantifies valgus corrective moments generated by a knee orthosis during gait using an integrated six-axis force sensor. This approach advances the biomechanical understanding of orthotic function and emphasizes the influence of structural parameters on corrective performance. The findings demonstrate that modifying the medial lower-leg strut length alters the generated corrective moment, indicating that orthotists can customize orthosis design to more effectively meet individual patient needs more effectively. Collectively, these insights expand theoretical knowledge and offer actionable guidance for clinical practice.

Several limitations should be acknowledged. First, the generalizability of the findings is constrained by participant selection; only individuals with unilateral left-sided knee OA, whose contralateral right knees were confirmed to be radiographically normal, were included to standardize biomechanical testing, orthosis fitting, marker placement, and the interpretation of EKAM and orthosis-generated corrective moments within a unified coordinate system. Consequently, caution is warranted when extrapolating these results to individuals with right-sided or bilateral knee OA. Furthermore, the sample size was relatively small, particularly for correlation analyses, and factors such as limb dominance, subtle side-to-side gait asymmetry, and compensatory gait strategies were not directly assessed. In addition, potential confounding factors such as sex, age, and leg length were not explicitly controlled. These factors were not included as covariates because the limited sample size of this pilot study could have reduced the stability of multivariable statistical models.

Second, the potential impact of prior brace experience and wearing-time-related adaptation was not comprehensively characterized. While participants’ previous use of braces was validated, detailed information regarding the duration and frequency of brace wear was not collected. Therefore, possible effects of wearing time, adaptation to the orthosis, and learning effects could not be evaluated. Furthermore, the study focused solely on the immediate biomechanical effects of orthosis use, without evaluating longer-term neuromuscular adaptations or clinical outcomes.

Third, orthosis design parameters and measurement-related factors were not fully standardized. Only medial strut length was examined as the primary structural parameter, whereas other orthotic characteristics, such as strap tension, brace stiffness, interface compliance, and alignment accuracy, were not quantitatively controlled. Furthermore, although the six-axis force sensor had adequate rated capacity and factory calibration, potential dynamic measurement uncertainties—such as sensor drift, crosstalk, and alignment errors—were not independently quantified.

Fourth, the biomechanical interpretation of the underlying mechanism remains inferential. Although a longer strut is expected to increase the effective moment arm and thereby generate a greater valgus corrective moment, neither the effective moment arm nor associated alignment changes were directly measured in vivo. Moreover, the reduction in EKAM was limited to the loading response phase, indicating that the unloading effect of the orthosis may be temporally limited. As such, a causal relationship between the orthosis-generated corrective moment and EKAM reduction could not be established.

Finally, walking speed was neither prescribed nor included as a covariate in the statistical analyses. Because walking speed can influence the magnitude of EKAM, the observed changes in EKAM may partially reflect speed-related gait adaptations. Therefore, the independent mechanical contribution of the orthosis-generated corrective moment cannot be completely separated from the potential effects of walking speed.

## Conclusions

This study examined the impacts of medial lower-leg strut length on the valgus corrective moment generated by orthoses and on EKAM during ambulation. The orthosis with a 192 mm strut produced larger valgus corrective moments compared with that of the 152 mm strut across the entire stance phase. This outcome indicates that increasing the moment arm—from the distal end of the strut to the knee joint center—can enhance the generated moment under equivalent loading conditions. Furthermore, a significant negative correlation was observed between the valgus corrective moment generated by the orthosis and EKAM, with the strongest association observed under the 192 mm strut condition (ρ = −0.694, *p* < 0.01). These results provide quantitative evidence that orthotic design influences EKAM, particularly during the loading response phase. Notably, the biomechanical unloading effect was phase-specific and should not be interpreted as a uniform reduction across the entire stance phase.

Stride length did not differ significantly across the no-orthosis, 152 mm strut orthosis, and 192 mm strut orthosis conditions. However, gait speed was slightly reduced in the 192 mm strut orthosis case (*p* < 0.05), likely reflecting increased dynamic constraint imposed by the longer strut, which may have prompted implicit changes in cadence. Medial lower-leg strut length was associated with valgus corrective moment magnitude and phase-specific EKAM reduction differences, particularly during the loading response phase. These findings highlight the importance of medial strut length as a critical biomechanical design parameter and provide a quantitative basis for optimizing orthotic strategies in future research. Further clinical studies incorporating patient-reported outcomes and extended follow-up periods are necessary to elucidate the practical therapeutic implications of these biomechanical findings. This study provides an empirical foundation for the future design and evaluation of knee orthoses by demonstrating that structural variations can influence the corrective moment generated during gait. In the long term, these insights may facilitate the development of more evidence-based and personalized orthotic management strategies for patients with medial knee OA, enabling orthosis selection and adjustment tailored to individual biomechanical profiles. These types of advances may help enhance pain management, preserve ambulatory function, and support daily mobility in patients with knee OA. From a broader perspective, optimizing orthotic interventions may also help mitigate functional decline and reduce the societal burden associated with knee OA. Future studies should investigate additional design variables, such as overall orthosis stiffness, strap positioning, and material composition, and should evaluate the long-term clinical effectiveness of orthosis use in larger and more diverse populations with varying severities of knee OA and limb deformities.

## Supporting information

S1 File Anonymized individual-level dataset used in this study. The Excel file contains the minimal individual-level dataset required to reproduce the findings of this study.
(XLSX)
